# Hyperuricemia is associated with an increased prevalence of ventricular tachycardia and fibrillation in patients with ST-elevation myocardial infarction after primary percutaneous coronary intervention

**DOI:** 10.1186/s12872-022-02635-4

**Published:** 2022-04-26

**Authors:** Xianqing Hu, Shenwen Fu, Saibin Wang

**Affiliations:** 1 Department of Cardiovascular Medicine, Jinhua Municipal Central Hospital, Jinhua Hospital of Zhejiang University, No. 365, East Renmin Road, Jinhua, 321000 Zhejiang Province China; 2 Department of Respiratory Medicine, Jinhua Municipal Central Hospital, Jinhua Hospital of Zhejiang University, Jinhua, Zhejiang Province China

**Keywords:** Hyperuricemia, Ventricular tachycardia, Ventricular fibrillation, ST-segment elevation myocardial infarction

## Abstract

**Background:**

Little is known about the association between hyperuricemia and ventricular tachycardia and fibrillation (VT/VF) in patients with ST-segment elevation myocardial infarction (STEMI) undergoing primary percutaneous coronary intervention (PPCI).

**Methods:**

The data from a cohort of STEMI patients undergoing PPCI at our center from January 2013 to December 2018 were retrospectively analyzed. The endpoint of the study was the occurrence of VT/VF, including (1) non-sustained ventricular tachycardia (nsVT) on Holter monitoring; (2) sustained ventricular tachycardia (SVT)/VF on cardiac monitoring.

**Results:**

Of the 634 patients included in the study, 147 (23.2%) of them had hyperuricemia. The occurrence of VT/VF after PPCI was significantly higher in patients with hyperuricemia (19.0 vs. 9.4%, *p* = 0.001) compared with those without hyperuricemia. Hyperuricemia was associated with a significantly higher risk of VF/VT (odds ratio (OR) 2.11; 95% CI 1.11–4.03; *p* = 0.024). The strength of this association remained statistically after adjustments for age, sex, history of hypertension, estimated glomerular filtration rate, hypersensitive C reactive protein, plasma natrium, peak troponin I, fasting glucose, B-type natriuretic peptides and VT/VF in PPCI (adjusted odds ratio 2.73; 95% CI 1.19–6.27; *p* = 0.018).

**Conclusions:**

There is a significant association between hyperuricemia and increased prevalence of VT/VF in STEMI patients after PPCI, independently of multiple risk factors and potential confounders.

**Supplementary Information:**

The online version contains supplementary material available at 10.1186/s12872-022-02635-4.

## Background

Ventricular tachycardia and fibrillation (VT/VF) are fatal arrhythmias causing cardiac collapse in acute myocardial infarction (AMI). VT is a marked sudden cardiac death predictors after adjustment for age, diabetes, and left ventricular ejection fraction (LVEF) in AMI patients [1, 2]. Even in the PCI era, VT/VF is associated with higher in-hospital deaths in all patients with ST-segment elevation myocardial infarction (STEMI) and higher 5-year mortality in high-risk STEMI patients [3]. Although previous studies have indicated predictors for the occurrence of VT/VF including preprocedural and postprocedrual thrombolysis in myocardial infarction (TIMI) flow, total baseline ST-segment deviation, creatinine clearance and Killip class in patients undergoing primary percutaneous coronary intervention (PPCI) [4, 5], the potential preventable and reversible risk factors for VT/VF is still required.

Hyperuricemia is defined as a serum uric acid level > 7 mg/dl for men and > 6 mg/dl for women [6], and has been associated with a higher risk of coronary artery disease, AMI and increased cardiovascular mortality [7–10]. The correlation between hyperuricemia and arrhythmia has also been reported. Both Cross-sectional [6, 11] and prospective [12, 13] studies show an independent association between hyperuricemia and atrial fibrillation. Serum uric acid is a significant middle-term predictor of electrocardiographically diagnosed tachyarrhythmia in general population [14]. However, the evidence is sparse in terms of the relationship between hyperuricemia and VT/VF. The present study was aimed to investigate whether hyperuricemia was associated with the occurrence of VT/VF in STEMI patients undergoing PPCI.

## Methods

### Study population

This retrospective observational study enrolled consecutive STEMI patients undergoing PPCI at Jinhua Municipal Central Hospital from January 1, 2013 to December 30, 2018. Patients with missed data for serum uric acid were excluded. Hyperuricemia was defined as a serum uric acid level > 7 mg/dl for men and > 6 mg/dl for women. The study size was referred to the relevant research [15].

### Clinical and laboratory data

Information on demographics, symptoms, established cardiovascular risk factors, previous cardiovascular events, biochemical indicators, echocardiography, medication, periprocedural features, Holter and electrocardiogram monitoring, and in-hospital adverse cardiac events (VT/VF, death, acute heart failure, stent thrombosis, target lesion revascularization) were abstracted from Haitai 2.0 electronic medical record system.

Troponin I levels were measured every 4 h after admission until its peak value was identified. Other venous blood samples, obtained between 6 and 7 am after 14 h of fasting, were sent to the hospital laboratories. Serum levels of uric acid, potassium, natrium, chlorinum, creatinine, total cholesterol, high-density lipoprotein cholesterol (HDL-C), low-density lipoprotein cholesterol (LDL-C), triglycerides and plasma glucose were measured by using enzymatic methods with reagents supplied by Beckman Coulter Diagnostics on the AU5800 analyzer (Beckman Coulter Inc, USA). Hypersensitive C-reactive protein (hsCRP) levels were determined with a highly sensitive immunoturbidimetry-based assay (Goldsite Diagnostics Inc, China). Plasma B-type natriuretic peptides (BNP) and Serum troponin I were measured by chemiluminescence immunoassay on the Dimension® EXL™ with LM Integrated Chemistry System (Siemens Inc, Germany). Estimated glomerular filtration rate (eGFR) was calculated with MDRD formula. The laboratory technicians were blinded to the objective of the research.

### Electrocardiogram data

All patients received cardiac monitoring for at least 96 h after admission and 597 patients received 24-h Holter monitoring after PPCI. Treatment with beta-blockers or other antiarrhythmic agents was continued during monitoring. Evaluation of Holter monitoring was performed twice by two professional physicians who were blinded to the aim of the study.

### Treatment

PPCI procedures were performed according to Chinese guidelines for percutaneous coronary intervention. Before the procedure, all patients received 300 mg aspirin combined with either 300–600 mg clopidogrel or 180 mg ticagrelor as loading dose. Use of thrombus aspiration, direct stenting, postdilation, glycoprotein IIb/IIIa inhibitors and anti-arrhythmia agents in PPCI was left to the physicians’ discretion. The Academic Research Consortium High Bleeding Risk criteria was used for bleeding risk stratification [16].

### Study outcome and definition

The outcome of interest was the occurrence of VT/VF, including (1) non-sustained ventricular tachycardia (nsVT) on Holter monitoring; (2) sustained ventricular tachycardia (SVT)/VF on cardiac monitoring. nsVT was defined as three or more consecutive ventricular premature beats with a rate of at least 120 beats per minute and persisting less than 30 s. SVT was defined as VT lasting longer than 30 s or requiring termination because of hemodynamic collapse. VF was characterized as a rapid, irregular, dysmorphic pattern with no clearly defined QRS complex resulting in rapid hemodynamic collapse. The diagnosis of STEMI was confirmed according to the third universal definition of myocardial infarction [17]. No-reflow was defined as thrombolysis in myocardial infarction (TIMI) flow < 3 after stent implantation [18].

### Statistical analysis

Continuous variables following normal distribution were expressed as mean ± standard deviation (SD) and those following non-normal distribution as median (interquartile range). Categorical variables were expressed as percentage. The normally distributed variables were compared by Student’s *t*-test and non-normal distribution variables by the Mann–Whitney *U* test. The Chi squared test were used for categorical variables. Variables were selected into the binary logistic regression model to identify the independent risk factors of VT/VF on the basis of their significance in univariable analyses. Three forced-entry logistic regression models were performed: an unadjusted model; a model adjusted for age and sex (model 1); and a model further adjusted for potential confounding factors including history of hypertension, eGFR, hsCRP, serum natrium, peak troponin I, BNP, plasma fasting glucose and VT/VF in PPCI (model 2). Statistical significance was set at *p* < 0.05. All statistical analysis were carried out by SPSS software (version 13.0, SPSS Inc. Chicago, IL, USA).

## Results

A total of 640 STEMI patients receiving PPCI were identified in electronic database. After excluding 6 patients with missing data for serum uric acid, 634 patients (503 males and 131 females) were finally included in the analysis. Of the 634 patients included, 147 of them had hyperuricemia. Information on demographics, clinical features, and treatments of patients stratified by the presence of hyperuricemia are shown in Table 1. Compared with those without hyperuricemia, patients with hyperuricemia were older, had a greater prevalence of hypertension and cardiogenic shock (Killip class IV), higher value of hsCRP, serum natrium, peak troponin I, BNP and plasma fasting glucose. Patients with hyperuricemia also had lower values of eGFR and LVEF, less likely to be treated with statin. There was no significant difference between groups with respect to the prevalence of VT/VF before PPCI.

**Table 1:** Clinical and biochemical characteristics of the patients with STEMI stratified by the presence of hyperuricemia

Characteristics	Patients with hyperuricemia	Patients without hyperuricemia	*p* value
	(n = 147)	(n = 487)	
	Serum uric acid 8.9 (7.1–8.9) mg/dl	Serum uric acid 5.0 (4.1–5.7) mg/dl	
*Demographic characteristics*			
Age (year)	65.6 ± 15.1	62.0 ± 13.1	0.003
Male	114 (77.6)	389 (79.9)	0.542
Hypertension	97 (66.0)	265 (54.4)	0.013
Diabetes mellitus	27 (18.4)	87 (17.9)	0.889
Dyslipidemia	4 (2.7)	12 (2.5)	0.772
Current smoking	71 (48.3)	278 (57.1)	0.061
Previous myocardial infarction	6 (4.1)	11 (2.3)	0.246
Previous revascularization	5 (3.4)	19 (3.9)	0.781
Previous stroke	15 (10.2)	27 (5.5)	0.046
*Clinical presentation*			
SVT/VF before PPCI	8 (5.4)	19 (3.9)	0.417
Cardiogenic shock at admission	42 (28.6)	69 (14.2)	0.000
hsCRP (mg/L)	8.0 (3.6–19.8)	5.0 (2.0–16.0)	0.002
Serum potassium (mmol/L)	4.0 ± 0.4	4.0 ± 0.5	0.220
Serum natrium (mmol/L)	139.5 ± 4.1	138.6 ± 3.3	0.016
Serum chlorinum (mmol/L)	105.5 ± 7.7	105.1 ± 4.00	0.452
eGFR_MDRD_ (ml/min/1.73m^2^)	57.1 ± 20.2	80.2 ± 22.2	0.000
Fasting glucose (mmol/L)	8.2 ± 3.5	7.1 ± 2.8	0.001
HbA1c (%)	6.4 ± 1.5	2.7 ± 1.7	0.540
Peak troponin I (ng/ml)	67.6 (22.8–153.2)	42.8 (17.9–87.0)	0.009
BNP (pg/ml)	1149.5 (302.0–3376.5)	709.5 (254.3–1698.8)	0.003
Triglycerides (mmol/L)	1.7 ± 1.2	1.5 ± 1.1	0.056
Total cholesterol (mmol/L)	4.3 ± 1.3	4.2 ± 1.1	0.336
HDL-C (mmol/L)	1.0 ± 0.3	1.0 ± 0.3	0.133
LDL-C (mmol/L)	2.9 ± 1.0	2.8 ± 0.8	0.628
LVEF (%)	57.5 ± 11.4	60.1 ± 9.1	0.018
*Medical treatment after PPCI*			
Aspirin	146 (99.3)	486 (99.8)	0.410
Clopidogrel	135 (91.8)	462 (94.9)	0.170
Ticagrelor	11 (7.5)	25 (5.1)	0.281
Beta-blockers	91 (61.9)	328 (67.4)	0.222
Statin	138 (93.9)	485 (99.6)	0.000
ACEI/ARB	98 (66.7)	333 (68.4)	0.697
Aminodarone	21 (14.3)	48 (9.9)	0.131
Lidocaine	14 (9.5)	28 (5.7)	0.107

Sample size, n = 634. Data are expressed as mean ± OR, number of patients (percentage) or median (range)

STEMI, ST-segment elevation myocardial infarction; SVT, sustained ventricular tachycardia; VF, ventricular fibrillation; LVEF, left ventricular ejection fraction; PPCI, primary percutaneous coronary intervention; hsCRP, hypersensitive C reactive protein; eGFR, estimated glomerular filtration rate; BNP, B-type natriuretic peptides; HDL-C, high density lipoprotein cholesterol; LDL-C, low density lipoprotein cholesterol; ACEI, angiotensin converting enzyme inhibitor; ARB, angiotensin receptor blocker

Table 2 shows the infarct-related artery of patients with hyperuricemia was more likely to be left main but less likely left circumflex. Patients with hyperuricemia had higher prevalence of no-reflow and VT/VF in PPCI compared with those without hyperuricemia. There was no remarkable difference between the two groups related to the number of diseased vessels, TIMI flow grade 0 before PPCI, total ischemic time, diameter and total length of stents and recovery of TIMI flow grade 3 after PPCI. Predilation before stenting and thrombus aspiration were performed in most patients. Glycoprotein IIb/IIIa inhibitor was used in about one third of the patients.

**Table 2:** Angiographic and procedural finding of the patients with STEMI stratified by the presence of hyperuricemia

Characteristics	Patients with hyperuricemia	Patients without hyperuricemia	*p* value
	(n = 147)	(n = 487)	
	Serum uric acid 8.9 (7.1–8.9) mg/dl	Serum uric acid 5.0 (4.1–5.7) mg/dl	
*Angiographic*			
IRA			
Left anterior descending	79 (53.7%)	244 (50.1%)	0.439
Left circumflex	8 (5.4%)	69 (14.2%)	0.005
Right coronary artery	57 (38.8%)	175 (35.9%)	0.531
Left main	5 (3.4%)	0 (0%)	0.001
Two IRAs	2 (1.4%)	1 (0.2%)	0.136
ST-related myocardial infarction	3 (2.0%)	11 (2.3%)	1.000
Diseased vessels			
1	37 (25.2%)	136 (27.9%)	0.511
2	50 (34.0%)	154 (31.6%)	0.586
3	60 (40.8%)	197 (40.5%)	0.937
TIMI 0 flow before PPCI	107 (72.8%)	327 (67.1%)	0.197
*Procedural*			
Total ischemic time (hour)	6.0 (3.0–12.0)	6.0 (4.0–12.0)	0.407
Thrombus aspiration	108 (73.5%)	329 (67.6%)	0.175
Glycoprotein IIb/IIIa inhibitor	47 (32.0%)	154 (31.6%)	0.936
Predilation	131 (89.1%)	414 85.0%)	0.123
Predilation balloon diameter (mm)	2.3 ± 0.3	2.3 ± 0.3	0.696
Predilation balloon length (mm)	15.2 ± 2.9	15.7 ± 3.0	0.120
Stent number	1.3 ± 0.5	1.2 ± 0.5	0.116
Stent diameter (mm)	3.2 ± 0.4	3.2 ± 0.6	0.931
Stent length (mm)	32.4 ± 15.5	30.1 ± 16.6	0.151
Postdilation	58 (39.5%)	195 (40.0%)	0.965
Postdilation balloon diameter (mm)	3.3 ± 0.5	3.4 ± 0.4	0.743
Postdilation balloon length (mm)	12.7 ± 2.6	12.8 ± 2.4	0.839
Postdilation pressure (Atm)	16.6 ± 4.2	16.9 ± 3.2	0.665
Intra-aortic balloon pump	7 (4.8%)	8 (1.6%)	0.055
SVT/VF during PPCI	6 (4.1%)	6 (1.2%)	0.037
No-reflow	9 (6.1%)	12 (2.5%)	0.036
TIMI 3 flow after PPCI	144 (98.0%)	482 (98.0%)	0.396

Sample size, n = 634. Data are expressed as mean ± SD, number of patients (percentage) or median (range)

STEMI, ST-segment elevation myocardial infarction; IRA, infarct-related artery; ST, stent thrombosis; TIMI, thrombolysis in myocardial infarction; PPCI, primary percutaneous coronary intervention; SVT, sustained ventricular tachycardia; VF, ventricular fibrillation

Table 3 indicates that the occurrence of VT/VF after PPCI was significantly higher in patients with hyperuricemia (19.04 vs. 9.44%, *p* = 0.001). Paralleled results were shown in terms of nsVT by Holter monitoring and SVT/VF by cardiac monitoring (15.64 vs. 8.62%, *p* = 0.003 and 4.08 vs. 0.82%, *p* = 0.003 respectively). Notably, the prevalence of death and acute heart failure before discharge were remarkably increased in patients with hyperuricemia than those with normal serum uric acid levels.

**Table 3:** nsVT, SVT/VF and in-hospital clinical outcomes of the patients with STEMI stratified by the presence of hyperuricemia

	Patients with hyperuricemia	Patients without hyperuricemia	*p* value
	(n = 147)	(n = 487)	
	Serum uric acid 8.9 (7.1–8.9) mg/dl	Serum uric acid 5.0 (4.1–5.7) mg/dl	
*Ventricular tachycardia*	28 (19.0%)	46 (9.4%)	0.001
nsVT by Holter monitoring	23 (15.6%)	42 (8.6%)	0.003
SVT/VF by cardiac monitoring	6^a^ (4.1%)	4 (0.8%)	0.013
*Clinical outcomes*			
Death	10 (6.8%)	5 (1.0%)	0.000
Acute heart failure	25 (17.0%)	29 (6.0%)	0.000
Stent thrombosis	0 (0%)	0 (0%)	
Target lesion revascularization	0 (0%)	0 (0%)	

Sample size, n = 634. Data are expressed as number of patients (percentage)

nsVT, non-sustained ventricular tachycardia; SVT, sustained ventricular tachycardia; VF, ventricular fibrillation; STEMI, ST-segment elevation myocardial infarction

^a^One patient developed both nsVT on Holter and SVT on cardiac monitoring

Fifteen variables were selected into the binary logistic regression model to identify the independent predictor of VT/VF according to their significance in univariable analyses (Additional file 1: Table S1). In unadjusted logistic regression analysis, hyperuricemia was associated with an approximately twofold higher risk of VF/VT (unadjusted OR 2.11; 95% CI 1.11–4.03; *p* = 0.024). This association strengthened after adjusting for age and sex (OR 2.45; 95% CI 1.34–4.54; *p* = 0.004, model 1). Notably, the strength of this association remained statistically after additional adjustment for history of hypertension, eGFR, hsCRP, serum natrium, peak troponin I, BNP, plasma fasting glucose and VT/VF in PPCI (OR 2.73; 95% CI 1.19–6.27; *p* = 0.018, model 2). It meant that hyperuricemia increased the absolute risk of VT/VF by 173% in STEMI patients. In this regression model, other independent predictors of prevalent VT/VF included history of hypertension and LDL-C (Table 4).

**Table 4:** Association between hyperuricemia and the risk of VT/VF in STEMI patients undergoing PPCI

Logistics regression models	OR	95% CI	*p* value
*Hyperuricemia*			
Unadjusted model	2.11	1.11–4.03	0.024
Adjusted model 1	2.45	1.34–4.54	0.004
Adjusted model 2	2.73	1.19–6.27	0.018
*Other independent risk factors in model 2*			
Hypertension	2.31	1.08–4.97	0.032
LDL-C	1.76	1.11–2.80	0.017

VT, ventricular tachycardia; VF, ventricular fibrillation; STEMI, ST-segment elevation myocardial infarction; PPCI, primary percutaneous coronary intervention; LDL-C, low density lipoprotein cholesterol

## Discussion

The present study showed that hyperuricemia was significantly associated with an increased prevalence of VT/VF in STEMI patients after PPCI. The significance of this association persisted after adjustment for multiple established risk factors and potential confounders. To our limited knowledge, this study may be the first report concerned the association between hyperuricemia and VT/VF in STEMI patients.

As early as 1985, McDonald et al. reported a relationship between serum uric acid and ventricular ectopy [19]. The association between increased serum uric acid levels and the occurrence of VT (defined as 5 or more consecutive ventricular beats on a 24-h ECG recording) in 167 patients with left ventricular hypertrophy was also confirmed [15]. Most recently, one observational study showed allopurinol use and use duration of more than 6 months were independently associated with a lower risk of ventricular arrhythmia in 28,775 gout patients, regardless of whether receiving anti-arrhythmic and cardio-protective medications or not [20]. The beneficial effect of allopurinol may come from uric acid decrease, which affects pathophysiologic pathways attenuating the vulnerability of the ventricular myocardium to ventricular arrhythmia, but not via an unidentified direct anti-arrhythmic action (in which case the results would have been apparent immediately upon treatment initiation), hinting a potential association between hyperuricemia and ventricular arrhythmia [21]. In the present study focusing on the most vulnerable group to ventricular arrhythmia [22], the association between hyperuricemia and the prevalence of VT/VF remained statistically significant after adjusting for potential confounding factors including age, sex, history of hypertension, eGFR, hsCRP, serum natrium, peak troponin I, BNP, plasma fasting glucose and VT/VF in PPCI, suggesting hyperuricemia is not merely an innocent bystander but may be implicated in the development of VT/VF in STEMI patients.

Although it is fairly certain that there is inner relationship between hyperuricemia and ventricular tachycardia, but evidence of causation is still needed. Ventricular arrhythmia has a complex pathophysiologic background and has been previously attributed to inflammatory states [23]. The generation of reactive oxygen species can contribute to induction of arrhythmias, via multiple mechanisms, including the alteration of cardiac ionic channels [24] and cardiac cell death associated ventricular dysfunction [25]. It is known that elevated serum uric acid can enhance the inflammatory response after STEMI [26]. But it is controversial whether hyperuricemia is only a marker of oxidative stress or directly induce VT/VF in STEMI patients. Use of allopurinol significantly decreased the inducibility of ventricular tachycardia and ventricular fibrillation in infarcted rats by down-regulating sympathetic innervation through a superoxide-dependent pathway, but the uricosuric agent benzbromarone had no beneficial effects on oxidative stress, sympathetic hyperinnervation or arrhythmia vulnerability at the similar levels of uric acid [27], indicating that uric acid functions only as an indicator of xanthine oxidase (XO) activity and is not directly involved in the arrhythmogenic process. However, there are well-established interspecies differences in intrinsic levels of myocardial XO activity [28]. Rats have relatively high levels of myocardial XO activity during myocardial ischemia, whereas the activity in humans is comparatively low [29]. Thus, finding from animal study cannot necessarily be generalized to species with comparatively low activities of XO. Meanwhile, hyperuricemia is associated with impaired myocardial reperfusion and greater infarct size [30, 31], which associated with higher rates of ventricular arrhythmias [32, 33]. The present study confirmed these data: patients with hyperuricemia presented with increased troponin I levels, risk of no-reflow and worse LVEF.

VT/VF are a major cause of morbidity and mortality in STEMI patients. Even modest correlations between hyperuricemia and VT/VF, and modest therapeutic effects could have significant clinical impact, given the high prevalence of STEMI. Compared with other established predictors for the occurrence of VT/VF in patients undergoing PPCI, such as pre-PCI TIMI flow, total baseline ST deviation, creatinine clearance and Killip class [4, 5], hyperuricemia is a preventable and reversible risk factor which may be a potential therapeutic target for subjects vulnerable to VT/VF, including patients with coronary artery disease, hypertension or cardiomyopathy [34].

Postprocedrual TIMI flow grade less than 3 did not increase the risk of VT/VF in the present study. The higher rate of TIMI 3 flow after PPCI compared with the previous study [4] (98.0% vs. 87.0%) might dilute the adverse effect of poor TIMI flow on VT/VF.

Several limitations of this study should be mentioned. First, the relatively small sample size and short-term follow-up make it limited to explore the prognostic implication of hyperuricemia. Second, it is a single-center, retrospective study that may lead to patient selection bias. Third, 37 patients did not receive 24-h Holterr monitoring, although it was comparable between the two groups. Fourth, ST-segment deviation and resolution is not included in the present study. Although 98% of the patients received TIMI 3 flow, which only reflecting epicardial rather than myocardial perfusion. Fifth, serum uric acid and creatinine levels, which change over time in the first days after AMI, may not reflect true baseline values because all blood samples were obtained early in the morning regardless of patient arrival time.

## Conclusions

The present study indicated a significant association between hyperuricemia and increased prevalence of VT/VF in STEMI patients after PPCI. Further basic research to establish a causal link between hyperuricemia and VT/VF, as well as translational studies and clinical trials to investigate the therapeutic implications of such a relationship is needed.

## Supplementary Information

**Additional file 1**. Univariate and multivariate analysis of risk factors for VT/VF in STEMI patients undergoing PPCI.

## Data Availability

The datasets generated and/or analyzed during the current study are not publicly available for protecting study participants, privacy, however data is available with corresponding author (Xianqing Hu) if data can be made available on reasonable request.

## Abbreviations

VT/VF: Ventricular tachycardia and fibrillation
STEMI: ST-segment elevation myocardial infarction
PPCI: Primary percutaneous coronary intervention
nsVT: Non-sustained ventricular tachycardia
SVT: Sustained ventricular tachycardia
eGFR: Estimated glomerular filtration rate
hsCRP: Hypersensitive C reactive protein
BNP: B-type natriuretic peptides
AMI: Acute myocardial infarction
LVEF: Left ventricular ejection fraction
TIMI: Thrombolysis in myocardial infarction
HDL-C: High-density lipoprotein cholesterol
LDL-C: Low-density lipoprotein cholesterol
XO: Xanthine oxidase
VF: Ventricular fibrillation
ACEI: Angiotensin converting enzyme inhibitor
ARB: Angiotensin receptor blocker
IRA: Infarct-related artery
ST: Stent thrombosis
