# Supplementary material for: Hyperuricemia is associated with an increased prevalence of ventricular tachycardia and fibrillation in patients with ST-elevation myocardial infarction after primary percutaneous coronary intervention
Source: BMC Cardiovasc Disord. 2022 Apr 26;22:199. doi: 10.1186/s12872-022-02635-4 (PMC9044610; doi:10.1186/s12872-022-02635-4)
Supplement: Supplementary file 1 — Additional file 1. Univariate and multivariate analysis of risk factors for VT/VF in STEMI patients undergoing PPCI. [file 12872_2022_2635_MOESM1_ESM.docx]

**Supplemental Table 1. Univariate and multivariate analysis of risk factors for VT/VF in STEMI patients undergoing PPCI**

| Variables | Univariate analysis | | | Multivariate analysis | | |
| --- | --- | --- | --- | --- | --- | --- |
|  | OR | 95% CI | p | OR | 95% CI | p |
| Previous stroke | 1.88 | 0.83-4.22 | 0.134 | 2.32 | 0.88-6.09 | 0.088 |
| Cardiogenic shock at admission | 1.48 | 0.82-2.66 | 0.188 | 1.67 | 0.75-3.73 | 0.209 |
| Hyperuricemia | 2.26 | 1.35-3.76 | 0.001 | 2.11 | 1.11-4.03 | 0.024 |
| Total cholesterol | 1.20 | 0.95-1.52 | 0.135 | 1.47 | 0.60-3.61 | 0.401 |
| LDL-C | 1.25 | 0.93-1.68 | 0.139 | 1.02 | 0.335-3.22 | 0.970 |
| LVEF | 1.02 | 0.99-1.04 | 0.155 | 1.01 | 1.00-1.04 | 0.609 |
| Beta-blockers | 1.56 | 0.90-2.70 | 0.111 | 1.49 | 0.75-2.94 | 0.256 |
| Left anterior descending | 1.48 | 0.90-2.42 | 0.119 | 1.27 | 0.63-2.55 | 0.510 |
| Left circumflex | 0.38 | 0.14-1.07 | 0.059 | 0.61 | 0.19-1.98 | 0.410 |
| Two diseased vessels | 1.51 | 0.92-2.49 | 0.101 | 0.97 | 0.51-1.83 | 0.920 |
| Glycoprotein Ⅱb/Ⅲa inhibitor | 1.45 | 0.88-2.40 | 0.141 | 1.44 | 0.75-2.75 | 0.271 |
| Predilation balloon length | 1.09 | 0.99-1.19 | 0.075 | 1.06 | 0.94-1.18 | 0.346 |
| Stent number | 1.82 | 0.95-3.49 | 0.072 | 1.26 | 0.50-3.19 | 0.620 |
| Stent length | 1.02 | 1.00-1.04 | 0.080 | 1.01 | 0.98-1.04 | 0.626 |
| SVT/VF during PPCI | 2.59 | 0.68-9.78 | 0.155 | 2.63 | 0.58-11.97 | 0.211 |

LDL-C=low density lipoprotein cholesterol; LVEF=left ventricular ejection fraction; PPCI=primary percutaneous coronary intervention; STEMI=ST-segment elevation myocardial infarction; SVT=sustained ventricular tachycardia; VF=ventricular fibrillation; VT=ventricular tachycardia.
